# An Investigation on the Hardness of Polylactic Acid Parts Fabricated via Fused Deposition Modeling

**DOI:** 10.3390/polym14142789

**Published:** 2022-07-08

**Authors:** Yu-Shan Zeng, Ming-Hsien Hsueh, Chao-Jung Lai, Te-Ching Hsiao, Chieh-Yu Pan, Wen-Chen Huang, Chih-Hao Chang, Shi-Hao Wang

**Affiliations:** 1 Department of Mechanical Engineering, National Kaohsiung University of Science and Technology, Kaohsiung 807618, Taiwan; tzen010@gmail.com (Y.-S.Z.); hugo@nkust.edu.tw (T.-C.H.); 2 Department of Industrial Engineering and Management, National Kaohsiung University of Science and Technology, Kaohsiung 807618, Taiwan; shwang@nkust.edu.tw; 3 Department of Fashion Design and Management, Tainan University of Technology, Tainan 710302, Taiwan; 4 Department and Graduate Institute of Aquaculture, National Kaohsiung University of Science and Technology, Kaohsiung 811213, Taiwan; panjade@nkust.edu.tw; 5 Department of Information Management, National Kaohsiung University of Science and Technology, Kaohsiung 824005, Taiwan; wenh@nkust.edu.tw; 6 Department of Marketing and Distribution Management, National Kaohsiung University of Science and Technology, Kaohsiung 824005, Taiwan; joechang@nkust.edu.tw

**Keywords:** 3D printing, fused deposition modeling, PLA, UV curing, hardness, printing angle, raster angle

## Abstract

This paper investigated the hardness property of the fused deposition modeling (FDM)-printed PLA samples via different process parameters of printing and raster angles. The hardness data were sampled from the flat and edge surfaces of the samples. In addition, the effect of hardness characters after the ultraviolet (UV) curing process was analyzed. Furthermore, this research found that the printing and raster angles significantly affected the hardness value of the PLA part, which slightly increased after the UV irradiation. Moreover, the results of this study will provide a reference for the field of FDM application.

## 1. Introduction

Fused deposition modeling (FDM) is one of the most popular and used technique in additive manufacturing (AM) due to its simple operation, low cost, and variety of materials [1,2,3,4]. The FDM method is popular due to its highly flexibility and process speed, but the durability of the printed material is the major concern, especially for products applicated in an outdoor environment [5]. The popular materials used in the FDM method include polylactic acid (PLA) [6], acrylonitrile butadiene styrene (ABS) [7,8], polyethylene terephthalate (PET) [9], polyethylene terephthalate glycol (PETG) [10,11], and others composite fibers [12,13,14]. The digital prototype of the part is usually created by CAD software and exported as an STL file. Then, the slicing software will slice the file and transfer it into a G-code format, which allows the FDM printer to read and manufacture the part. After the printer constructs the part completely, a certain part will be irradiated by UV light via a UV curing machine, a post-processing step commonly used in composite fiber material for stronger polymer chains and physical entanglements [15]. A large number of studies have been conducted to explore the processing parameters to optimize the manufacturing efficiency and the product mechanical properties (e.g., tensile, compressive, bending, and hardness) of the FDM process. Rajpurohit et al. [16] used different angles, layer thickness, and raster widths to explore the tensile properties of PLA printed parts. The results found that the 0° raster angle has the highest tensile strength. In addition, Liu et al. [17] analyzed the tensile and flexural behaviors of multiple PLA composite parts printed in different orientation and raster angles. The results indicated that the PLA part printed in an on-edge orientation with +45°/−45° raster angle has the highest tensile strength and modulus. Furthermore, Ding et al. [18] used the finite element simulation to explore the stiffness and strength of the specimens. The results showed that it is feasible to predict the stiffness and strength of FDM specimens using classical laminated plate theory and the Tsai–Wu failure criterion and that the 45° printing angle leads to specimens with maximized tensile properties. Numerical simulations show that changing the printing angle highly impacts the mechanical characteristic of the PLA part. Kumar et al. [19] investigated the mechanical properties of the PLA composite matrix and observed that the printing angle of 45° has the best condition on mechanical properties (flexural and pull out), which are dependent upon the hardness, surface porosity and surface roughness. On the other hand, Butt et al. [20] used the Hybrid Fused Deposition Modelling (H.F.D.M.) process to explore the mechanical characteristic of Cu/PLA composite. The experimental results indicated that the hardness value is greatly affected by surface roughness. Vicente et al. [21] used ABS material to analyze the effect of different nozzle diameters, layer thicknesses, infill densities, and raster angles on the mechanical properties. The results found that the nozzle diameter mostly affects the tensile strength and stiffness. In particular, a higher nozzle diameter increases the value of stiffness and tensile strength. Bedi et al. [22] reported the mechanical properties of the printed part for in-house prepared feedstock filament comprising of SiC/Al_2_O_3_ reinforced in recycled low-density polyethylene (LDPE) matrix with different particle sizes. The results showed that the raster angle has the maximum impact on the dimension accuracy and hardness of the printed part. Meanwhile, Harven et al. [23] investigated the effect of three different building thicknesses and two different layer orientations of PLA material on FDM printed samples. The Shore D test results found that the sample printed in horizontal orientation has higher hardness values and that the building orientation has a significant effect on the hardness of the printed material. Chohan et al. [24] used the process parameters of the combined FDM and vapor smoothing (VS) process to optimize ABS replicas for sustainability in biomedical applications. The ABS parts were put into a cooling chamber for pre-cooling process. The results showed that the hardness of the replica had been slightly increased by the maximum impact of the orientation angle and post-cooling time of ABS replicas. Valerga et al. [25] studied the effects of mechanical properties via different post-treatments of PLA printed samples. The results obtained obvious changes in the maximum stress (from 55 to 20 MPa), in elongation (from 3% to 260%), and in the hardness scale (Shore D to A). Felices et al. [26] investigated the effect of the addition of epoxysilane-treated wollastonite (ETW) to the mechanical and thermal properties of 3D-printed ABS via FDM; their hardness test results indicated that the addition of ETW increases the tensile strength, elastic modulus, and elongation at break of ABS by up to 46.6, 56.2, and 53.7%, respectively. The Shore D hardness increases with increasing ETW. Vishal et al. [27] used the FDM process to extrude silicon filled PLA biocomposite filaments with various weight percentages of sub-micrometric silicon particles (0, 1, 3, 5, and 7%) filled to improve the PLA performance. The results found that the interaction of silicon filler in the PLA matrix has a good load-carrying ability, and the PLA with 7% silicon biocomposite has the highest Shore D hardness, attributable to the silicon filler’s load-bearing capacity. Some researchers improve polymer via UV curing or irradiation process, which was considered as a method to increase the long-term stability of polymer [28]. Lyu et al. [15] added phosphine oxide (XBPO) in the PLA matrix, the results found that the maximum tensile strength of 3D printing specimens increased by 82.5% after UV process.

According to the past published studies, the effect of multiple process parameters on mechanical properties was widely discussed. We discussed the effects of different raster and printing angles, infill density and the UV process on tensile properties before [29,30]. However, since there is a lack of research exploring the impact of building direction on the hardness of the material, this research will follow with former research and further exploring the surface hardness properties from different printing angles of three different types (with X, Y, and Z axes) and three different raster angles of flat orientation specimens under low infill density conditions. In addition, the hardness properties of the front and the side surface will be investigated. Furthermore, the difference in the surface hardness before and after the UV curing process of the PLA printed part will be further analyzed as a simulation for the PLA part expose in an outdoor environment. Ultimately, the findings of this research could be a reference for the engineering application in the FDM process area.

## 2. Experimental Campaign

The FDM printer used in this paper is the X1E 3D printer (Infinity3DP, Kaohsiung, Taiwan), and a 0.4 mm diameter nozzle was selected. The process temperature and speed were set as 205 °C and 35 mm/s, respectively. Since most commercial product requires high production efficiency and short cycle time, the infill density of 10% and the layer thickness of 0.2 mm were thus chosen for shorter processing time. The fixed and controlling factors of the FDM process for fabricating the samples are shown in Table 1. The PLA filaments of Snow-White color (MIN-YAU, Taipei, Taiwan) are 1.75 mm in diameter. The digital model of the test samples was drawn by the Inventor software, and the output was exported in S.T.L. format. The KISSlicer and Cura software were used to set the process parameters of the samples and transform them into a G-code format for the printer to construct the samples. The purpose of this research is to analyze the hardness properties of PLA samples printed in two different types. One type is printed in certain angles (0°, 30°, 60°, and 90°) of three different axes (X, Y, and Z axes), are named as X-type, Y-type, and Z-type samples, respectively. These three types of samples are printed with 45° of raster angles. The other type is the sample that lies on the platform (X0/Y0 orientation) with three different raster angles (0°/90°, 30°/−60°, 45°/−45°), which are called the R-type samples. The schematic diagram of each sample is shown in Table 2; it should be noted that the specimens of X90/Z90 and Y0/Z0 were totally identical in the printing process, and therefore, they were tested with the same specimens. A total of two sets of identical samples were printed, of which one was irradiated by UV light to explore the effect of the UV curing process. After the FDM printer fabricated the samples completely, the samples with the UV curing process will be immediately sent to the UV curing machine for UV irradiation. The MultiCure 180 UV curing machine (XYZ Printing, New Taipei, Taiwan) was used with a wavelength of 365 nm and power intensity of 60%. The detailed specification of the curing machine are listed in Table 3. The rotational speed of the turntable was one revolution per minute. Then, the specimens were placed in a humidity-controlled box with 23 °C of room temperature to keep them dry and to prepare for the test. Each set of samples were irradiated by the curing machine for one hour.

**Figure 1:**
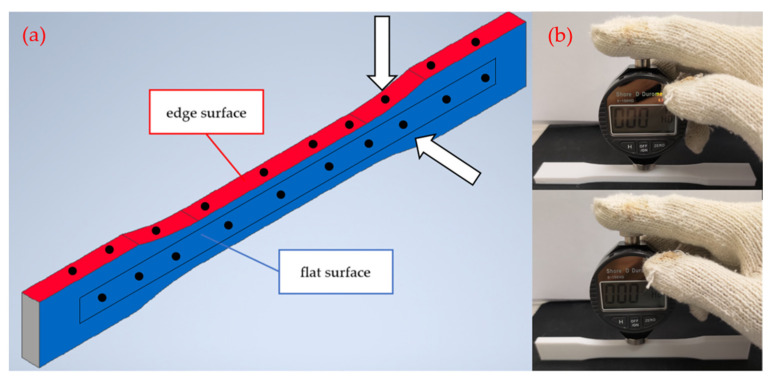
(**a**) The tested point and definition of the tested surface; (**b**) the setup of experiment.

**Figure 2:**
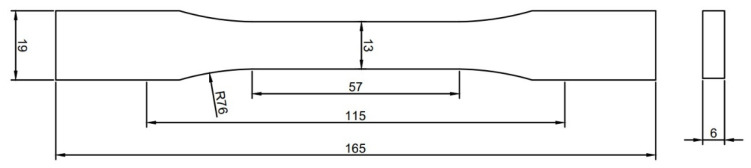
The specifications of the hardness test samples (mm).

A digital Shore D (HD) durometer (S.E.A.T Industry Technology, Kaohsiung, Taiwan) was used for the hardness test with the indentation needle of 0.1 mm diameter. According to the specification, the thickness of the tested sample should not be less than 6 mm. The hardness values were taken from the flat and the edge surfaces of the samples. Top/bottom layers are printed for three layers (0.6 mm), the perimeters are printed for three layers, i.e., 1.2 mm. The sample thickness of two different testing directions are 6 mm (flat-surface) and 13 mm (edge-surface), respectively. Figure 1a illustrates the definition of tested surface, the position of tested point and the load direction of durometer. The tested specimens were according to the Type I sample of ASTM D638 standard. The specification of the test samples is shown in Figure 2. For each type of sample, 5 identical specimens were printed, and the average value was picked. Then, 10 points from central axis of tested surface of each sample were tested, each point was tested 5 times and the extreme value was excluded. The change rate of Shore D hardness measurements of each surface before and after UV curing are analyzed and calculated by the following formula:

(1) $$\mathrm{Change}\;\mathrm{rate}=\frac{{\mathrm{HD}}_{UV}-{\mathrm{HD}}_{Original}}{{\mathrm{HD}}_{Original}}\times 100\%\;$$

where ${\mathrm{HD}}_{UV}$ and ${\mathrm{HD}}_{Original}$ represent the hardness value of the material with and without UV curing process, respectively.

## 3. Results

**Figure 3:**
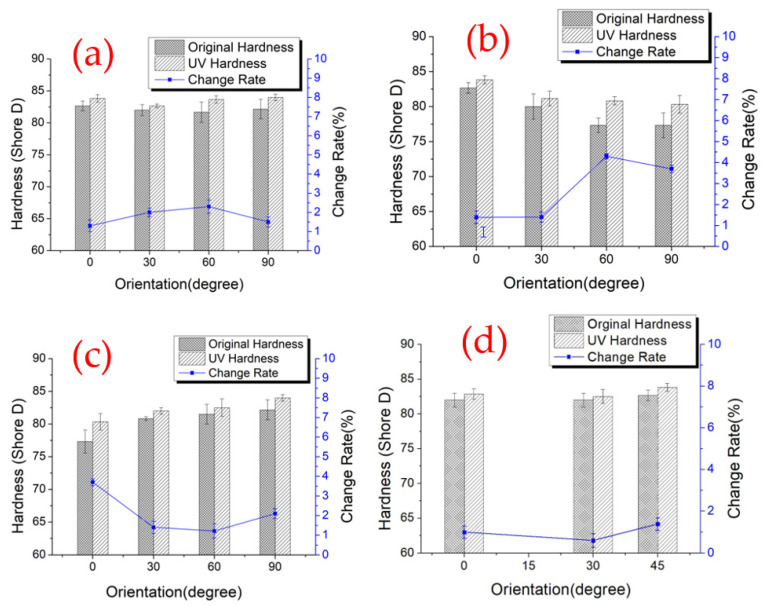
The hardness measurements of the flat surface: (**a**) X-type; (**b**) Y-type; (**c**) Z-type; (**d**) R-type.

The hardness measurements and the change rate after the UV curing process of the flat surface are shown in Figure 3, and the standard deviation of the change rate is indicated by the red line. The mean values and standard deviations of the original PLA and material after UV curing are shown in Table 4, respectively. For X-type samples, the flat-surface of X0° is the top layer, and the rest of the X-type samples are perimeter layers. Though the X0° sample has the thinnest layer thickness for the tested direction, there was no obvious trend for X-type samples before UV curing, but it could be observed that the hardness is slightly enhanced after the UV curing process. This is probably because the specimens are printed under low infill ratio, and the thickness of the printed specimens did not totally follow the specification of test standard; therefore, the measured hardness value did not show significant difference. The hardness trend of Y-type samples was decreased with the increase in the printing angle. The reason for the trend is probably because of the processing time of the sample surface. The Y90° samples took the longest processing time of the printer to fabricate the tested surface layer by layer because of its built direction. Meanwhile, the tested surface of the Y0° samples took the shortest processing time just for one layer, which promoted a better combination between fibers and resulted in a smoother surface. Additionally, it could also be seen, though, that the tested surface of the Y0° sample is the top layer of the printing process; otherwise, the tested surface of the Y90° sample is the perimeter layer, which has a thicker shell, but the longer fabrication time results in a rough surface and lower hardness value. To summarize, during the process of the Y30°, Y60°, and Y90° samples, the former layer was exposed in the air for a longer time after extrusion until the next layer overlap, which caused a poor combination between layers and led to a rough surface on the samples. Therefore, the tested surface has intensive valleys and peaks. When the indenter meets a valley, the obtained hardness value will be overestimated [20,31]. The above reason could be used to explain the Z-type samples, in which the hardness values were increased with the increasing printing angle, as shown in Figure 3c. Additionally, the tested surfaces of Z-type samples are all the perimeter layer, and the samples are basically tested with the same thickness, so the hardness property basically follows a linear relationship. Figure 3d shows the hardness measurements of the flat orientation samples with different raster angles. It can be seen that the raster angle has no significant impact on the hardness value of the flat samples. This is because changing the raster angles of the samples that lay down on the printing platform did not influence the roughness and the density of fibers of the tested surfaces much. Furthermore, the surface filaments were basically printed in the same width, and the hardness are all measured from the top layer with the same thickness; thus, the deviation of the hardness values fluctuates within ±1%.

**Figure 4:**
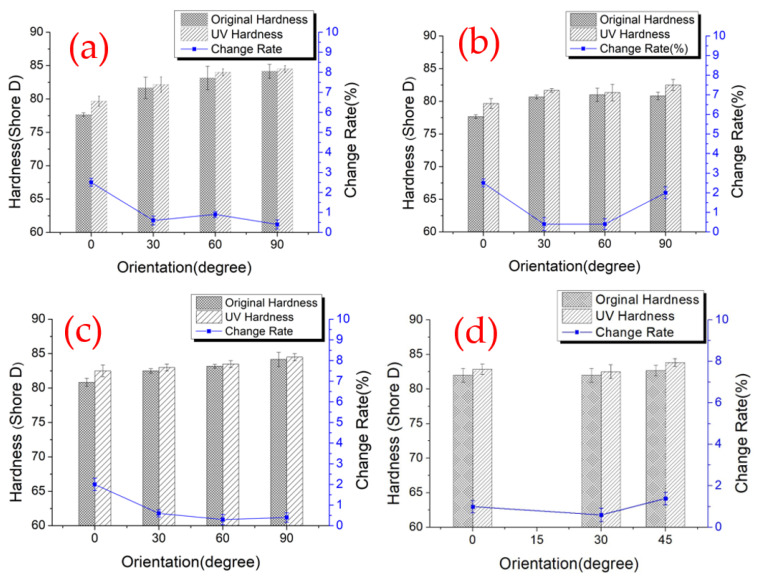
The hardness measurements of the side surface: (**a**) X-type; (**b**) Y-type; (**c**) Z-type; (**d**) R-type.

**Figure 5:**
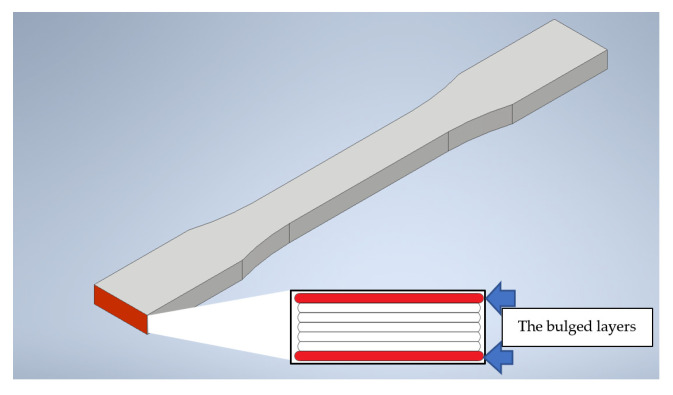
The schematic diagram of the bulged layers.

Figure 4 demonstrates the hardness value and the change rate after the UV curing process of the edge surface. The mean values and standard deviations of hardness value of original PLA and material after UV curing on edge surface are shown in Table 5, respectively. A slightly increasing trend in the X-type sample was recorded when the printing angle was over 0°. This phenomenon is possibly caused by the process orientation of the samples; when the printing angle is at 0°, the layers of the tested surface were vertical with the indentation needle. Thus, the first and last layer of the samples would be easily bulged, which was caused by different cooling times, as shown in Figure 5, resulting in the concave condition at the edge of the surface. In addition, the convex layers block the indentation needle, and it could not penetrate the tested surface deeply. Therefore, the hardness value is easily underestimated. The lower trend of the hardness value on the edge surface of the R-type samples can be explained similarly, despite the thicker perimeter shell for the test direction; this type of specimen lays on the platform during the printing process, and the concave surface also led the underestimation of the hardness measurement. The rest of the X-type samples did not show such a concave condition because their tested surfaces were built in a shorter time and did not totally paste on the platform. The smoother surface of the samples could better respond to the hardness properties of the original material. The same circumstances were also observed on the Y-type samples; the Y0° samples demonstrated the lowest hardness value, which is also because the thinnest tested surface of Y0° is in the Y-type samples; the rest of samples are all tested with the thicker perimeter layer. For the Z-type samples, the hardness measurements are all taken from the perimeter layer of the specimens, and it can be seen that the hardness values increased with the increase in the printing angle, due to the different printing directions. With the printing angle of 0° in the *Z*-axis, the layers with similar shapes were vertical to the indentation needle. Further, when the printing angle increased, the layer directions were more parallel to the press direction, which affected the result of the hardness value [32]. The hardness measurements of all sample types show a slight enhancement after the UV curing process, which may be due to the longer molecular chain after the UV irradiation enhanced the cross-linking point of the PLA materials [33]. In addition, the results shows that the anisotropy change rate in different printing orientations did not follow a linear relationship, which is because of the material with different process parameters. The dissimilar infill structure inside the samples led to a different UV absorption rate of the material, which resulted in inconsistent metallographic structures and an irregular change rate of each sample type.

## 4. Conclusions

In this research, the samples with two different process parameters were printed to explore their effect on the hardness property of the PLA material, including four different printing angles used on X, Y, and Z axes and five different raster angles used on flat orientation. The hardness values from two different surfaces of samples were tested and analyzed. The change in the hardness values before and after the UV curing process was also investigated. According to the experimental result, this study has the following findings:

1. Changing the printing angle affected the hardness property of the PLA material significantly. Most of the hardness trend of the flat surface basically followed the linear relationship with the printing angle except for the X-type samples. The flat surface of R type 90/0 samples demonstrated the highest hardness value of 83.3; on the contrary, the lowest value of the flat surface of 77.3 appears on Y60, Y90 and Z0 samples.
2. The hardness property of the edge surface increased with the printing angle, but the concave surface of the X0° and Y0° samples caused a lower hardness value due to their built orientation. The same situation was also replicated on flat-type samples, for which the 0/−90 sample has the lowest value of 77.3.
3. The hardness value collected from the flat surface did not show significant impact via the change in the raster angle.
4. The UV curing process enhanced the hardness property of the PLA material, while the extent of the change rate depended on the printing method of the tested sample.
5. The tested samples printed with a low infill ratio probably demonstrated an irregular trend on the hardness measurements results because of the empty inner structure, and a higher infill ratio should be used in future study for better stability in the experiment.

## Figures and Tables

**Table 1:** The fixed and controlling factor of FDM printer.

Fixed Factor	Value
Nozzle temperature	205 °C
Nozzle diameters	0.4 mm
Printing speed	35 mm/s
Infill density	10%
Platform temperature	60 °C
Layer thickness	0.2 mm
Filament diameters	1.75 mm
**Controlling factor**	**Value**
Printing axis	X, Y, Z
Printing angle	0°, 30°, 60°, 90°
Raster angle	0°/90°, 30°/−60°, 45°/−45°

**Table 2:** The schematic diagram and definition of the test sample.

Type	Name	Schematic Diagram
X−type	X0 (Y0)	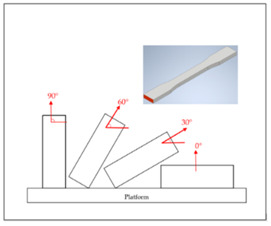
	X30	
	X60	
	X90 (Z90)	
Y−type	X0 (Y0)	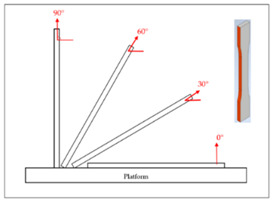
	Y30	
	Y60	
	Y90 (Z0)	
Z−type	Y90 (Z0)	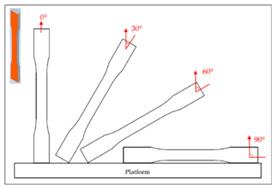
	Z30	
	Z60	
	X90 (Z90)	
R−type	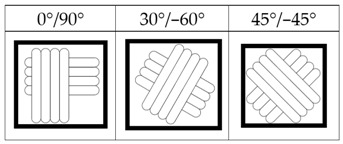	

**Table 3:** The fixed and controlling factors of UV curing machine.

Parameter	Value
Wavelength	365 nm
Rotation speed	1 rpm
Operating temperature	23 °C
Curing time	60 min

**Table 4:** The hardness (Shore D) measurement results on the flat surface.

Type of Specimens	Hardness (Original)	Hardness (UV)
X0	82.6 ± 0.7	83.8 ± 0.5
X30	80.7 ± 0.8	82.0 ± 0.2
X60	81.5 ± 1.6	82.5 ± 0.5
X90	82.2 ± 1.5	84.0 ± 0.5
Y0	82.6 ± 0.7	83.8 ± 0.5
Y30	80.0 ± 1.8	81.1 ± 0.2
Y60	77.3 ± 1.0	80.8 ± 0.5
Y90	77.3 ± 1.7	80.3 ± 0.2
Z0	77.3 ± 1.7	80.3 ± 1.0
Z30	80.8 ± 0.3	82.0 ± 0.5
Z60	81.5 ± 1.4	82.5 ± 1.3
Z90	82.2 ± 1.5	84.0 ± 0.5
0°/90°	82.1 ± 0.9	82.7 ± 0.7
30°/−60°	82.0 ± 1.0	82.4 ± 1.0
−45°/45°	82.6 ± 0.7	83.8 ± 0.5

**Table 5:** The hardness (Shore D) measurement results on the edge surface.

Type of Specimens	Hardness (Original)	Hardness (UV)
X0	77.6 ± 0.2	79.7 ± 0.7
X30	81.6 ± 1.6	79.6 ± 1.1
X60	83.1 ± 1.7	82.2 ± 0.4
X90	84.1 ± 1.0	84.5 ± 0.5
Y0	77.6 ± 0.2	79.7 ± 0.7
Y30	80.7 ± 0.2	81.7 ± 0.2
Y60	81.0 ± 0.2	81.3 ± 1.2
Y90	80.8 ± 0.5	82.4 ± 0.8
Z0	80.8 ± 0.5	82.4 ± 0.8
Z30	82.5 ± 0.5	83.0 ± 0.5
Z60	83.2 ± 0.2	83.5 ± 0.4
Z90	84.1 ± 1.0	84.5 ± 0.5
0°/90°	76.6 ± 0.5	79.8 ± 0.5
30°/−60°	78.8 ± 0.5	80.3 ± 1.0
−45°/45°	77.6 ± 0.2	79.7 ± 0.7
